# The 3-min appraisal of a meta-analysis

**DOI:** 10.4103/0019-5413.73652

**Published:** 2011

**Authors:** Nasir Hussain, Ammar Bookwala, Parag Sancheti, Mohit Bhandari

**Affiliations:** Orthopaedic Research Center, Division of Orthopaedic Surgery, Hamilton, Ontario; 1 Sancheti Institute for Orthopaedics and Rehabilitation, Pune, India

Meta-analysis can be defined as a quantitative method that mathematically combines the results from multiple studies covering the same overall topic, or the statistical pooling of the results of two or more similar studies.^1^,^2^ The results from meta-analyses can vary depending on the quality of the studies included and the methodological rigor used to select studies.^2^ We provide quick and simple criteria for critical appraisal, and a practical example illustrates the approach.

## KEY CRITERIA FOR CRITICAL APPRAISAL

Overall, three broad questions should be asked [Table 1].

**Table 1:** Questions that should be considered when critically appraising a meta-analysis

Are the results of the study valid?
Was the research question focused and clearly described?
Was the literature research systematic and reproducible?
Was the study selection process systematic?
Were the characteristics of the studies included presented?
Was a quality assessment of the studies included performed?
Were the statistical methods used to combine the studies reported?
Were the pooled studies homogenous?
Was publication bias assessed?
What are the results of the study?
What are the main results of the paper?
What is the significance of the results? Could they be due to chance?
What is the applicability of the results?
What is the applicability of the results?
Are the outcomes assessed clinically relevant?

## Are the results of the study valid?

One of the most important steps in critically appraising a meta-analysis is determining the methodological quality of the study design and the level of bias incorporated in the analysis. One key factor affecting the quality of meta-analysis is the quality of the studies that are included in the meta-analysis itself. One should remember that quality in equals quality out. Moreover, a meta-analysis should have a focused research question and a comprehensive literature search. 1 More importantly, the literature search should be systematic and reproducible.^1^,^2^ Readers should also be aware of publication bias, which refers to the increased probability of studies with positive results to be published.^1^,^3^

## What are the results?

The next step in critical appraisal is determining the main results and how they are expressed. Most times the results will be presented through a forest plot (or pictorial of the individual study findings). Overall combined results are usually presented as a relative risk or odds ratio when the outcomes are categorical (i.e. alive or dead, infection or no infection). When the results are continuous, as in a functional outcome score, the pooled results across many studies can be presented with standardized mean differences or effect sizes (difference in means divided by the standard deviation).

## What is the applicability of the results?

When critically appraising a meta-analysis the last step should be determining the clinical applicability of the results. Sometimes the results of a meta-analysis may show statistical significance, but may have no importance to practice. A simple questionnaire provided in Table 1 can be used as a quick reference guide for the critical appraisal of a meta-analysis.

## A PRACTICAL EXAMPLE: FIXED- VERSUS MOBILE-BEARING TOTAL KNEE REPLACEMENT

Smith and colleagues conducted a review of 33 studies assessing the outcomes of 3532 total knee replacements (TKRs).^4^ Analysis suggested that there was no significant difference in clinical or radiological outcomes and complication rates between fixed- and mobile-bearing TKRs.

*The research question*: To analyze the difference in clinical and radiological outcomes between fixed- and mobile-bearing TKRs.

Literature search, study selection, and quality analysis: The search strategy involved the use of Medline, CINAHL, AMED, and EMBASE to find included studies. Various search terms specific to the research question such as “knee AND fixed bearing OR mobile bearing” were formulated. Bias was accounted for by searching for the unpublished literature using the System for Information on Grey Literature (SIGLE). The selection criteria for all included and excluded studies are outlined and described. Furthermore, the quality of each study was methodologically and independently assessed by two reviewers using the Physiotherapy Evidence Database (PEDro) appraisal tool.

*Outcome measures and combination of studies*: The primary and secondary outcome measures are clearly indicated. Data were pooled using either pooled mean difference, standardised mean difference for continuous variables, or relative risk for dichotomous variables. These concepts were briefly described above.

*Main results and tests of significance*: Overall, it was found that there was no statistically significant difference with respect to functional, clinical, radiological outcomes or complication rates between fixed- and mobile-bearing TKR designs.

*Clinical relevance of results*: Since the results find no statistical significance it can be concluded that either fixed- or mobile-bearing TKR designs can be used. However, this should be done with caution since the study indicates limitations in the current evidence.

## CONCLUSION

Critical appraisal is an invaluable tool used in evidence-based medicine, and it is important for a clinician to determine the best quality evidence for practice. Along with the questionnaire provided in Table 1, a checklist is provided in Table 2 of the various items that should be included in a superior meta-analysis. Gaining a proper understanding the concepts described above and utilizing the tables provided will help in the evidence-based approach!

**Table 2:** The Do’s and Don’ts of a meta-analysis

Do’s	Don’t
Ensure that the research question is focused	Assume that the conclusions are relevant to clinical practice
Check if inclusion and exclusion criteria are present	Accept the conclusions without analyzing the methods
Ensure that the search is methodologically sound	Ignore the statistical results assume that they are methodologically sound
Ensure that level of bias is minimized	Incorporate bias in judgment of the meta-analysis
Check if quality analysis is performed	
Ensure that the tests for heterogeneity are performed	
Check the applicability of the results to clinical practice	
